# Diagnostic Performance of Prostate-Specific Membrane Antigen-Targeted Positron Emission Tomography in Patients Diagnosed with Different Types of Breast Cancer: A Systematic Review

**DOI:** 10.3390/ijms252111413

**Published:** 2024-10-24

**Authors:** Alessio Rizzo, Domenico Albano, Caterina Marchiò, Francesco Dondi, Manuela Racca, Francesco Bertagna, Francesco Fiz, Arnoldo Piccardo, Giorgio Treglia

**Affiliations:** 1 Department of Nuclear Medicine, Candiolo Cancer Institute, FPO—IRCCS, 10060 Turin, Italy; alessio.rizzo@ircc.it (A.R.); manuela.racca@ircc.it (M.R.); 2 Division of Nuclear Medicine, Università degli Studi di Brescia and ASST Spedali Civili di Brescia, 25123 Brescia, Italy; domenico.albano@unibs.it (D.A.); francesco.dondi@unibs.it (F.D.); francesco.bertagna@unibs.it (F.B.); 3 Pathology Unit, Candiolo Cancer Institute, FPO—IRCCS, 10060 Turin, Italy; caterina.marchio@ircc.it; 4 Department of Medical Sciences, University of Torino, 10060 Turin, Italy; 5 Nuclear Medicine Unit S.C., Galliera Hospital, 16128 Genoa, Italy; francesco.fiz@galliera.it (F.F.); arnoldo.piccardo@galliera.it (A.P.); 6 Clinic of Nuclear Medicine, Imaging Institute of Southern Switzerland, Ente Ospedaliero Cantonale, 6501 Bellinzona, Switzerland; 7 Faculty of Biology and Medicine, University of Lausanne, 1011 Lausanne, Switzerland; 8 Faculty of Biomedical Sciences, Università della Svizzera Italiana, 6900 Lugano, Switzerland

**Keywords:** breast cancer, PET, PSMA, neoangiogenesis, oncology, nuclear medicine

## Abstract

Recent research has proposed using positron emission tomography/computed tomography (PET/CT) along with the administration of prostate-specific membrane antigen (PSMA)-targeting radiopharmaceuticals to identify breast cancer (BC) lesions. An extensive literature review to investigate the possible diagnostic utility of PET/CT with PSMA-targeting radiopharmaceuticals in BC patients was performed. The research comprised different clinical scenarios, including both newly diagnosed BC patients and those who had experienced disease relapse. This updated systematic review encompassed six studies investigating the diagnostic efficacy of PSMA-targeted PET/CT in BC. Throughout all clinical settings investigated, the papers presented data demonstrating a modest diagnostic performance of PSMA-targeted PET/CT in different subtypes of BC. In this setting, PSMA-guided PET/CT showed slightly higher accuracy in patients diagnosed with triple-negative BC. Based on the current literature, PSMA-targeted PET/CT cannot be suggested as a diagnostic tool to assess BC extent in any clinical scenario. However, based on the PSMA expression observed in triple-negative patients, it can be proposed as a tool to evaluate whether BC patients could benefit from PSMA-targeting radioligand therapy.

## 1. Introduction

With an estimated 2.3 million new cases worldwide, breast cancer (BC) is currently one of the most common malignancies to be diagnosed and the fifth leading cause of cancer-related deaths [1]. With BC accounting for 11.7% of all cancer cases, it has surpassed lung cancer and other cancer types to become the world’s leading type of cancer. The World Health Organisation (WHO) and recent studies from 2021 and 2022 show that there were over 2.2 million BC diagnoses in women worldwide and over 500,000 deaths from the disease [2]. This statement means that approximately 15–16% of cancer-related deaths and 25–30% of cancer cases are related to BC [3]. There are significant differences in BC’s overall 5-year survival rate, ranging from 50% in less developed nations to 80% in developed countries [4].

The prevailing staging method for BC is the American Joint Committee on Cancer TNM system, which relies on seven criteria: tumour extent (T), dissemination to adjacent lymph nodes (N), presence of remote metastases (M), tumour grading (G), oestrogen receptor (ER) status, progesterone receptor status (PR), and human epidermal growth factor receptors (HER2) status [5]. Accordingly, BC has a broad disease spectrum [6] encompassing six main subtypes: luminal A (ER-positive), luminal B (ER-positive, HER2-enriched), HER2-enriched, basal-like, low-claudin, and normal-like BC. This classification is critical in therapeutic decision-making. ER-positive subtypes are hormone-sensitive forms which benefit from a targeted anti-hormonal approach and have an excellent overall prognosis [7]. Despite HER2 being a target for molecular therapies, its overexpression is associated with a worse prognosis. Finally, basal-like and low-claudin triple-negative BC (TNBC) subtypes are less differentiated and usually associated with poor clinical outcomes [7,8].

[^18^F]Fluorodeoxyglucose ([^18^F]FDG) positron emission tomography/computed tomography (PET/CT) is recommended for the initial whole-body work-up of patients diagnosed with locally advanced BC whenever distant metastases are suspected [9]. Additionally, [^18^F]FDG PET/CT can assess treatment efficacy in neoadjuvant and metastatic scenarios [10]. However, the accuracy of [^18^F]FDG PET/CT can be lower in differentiated forms, such as lobular BC [11]. Other approaches have been sought to complement BC’s molecular imaging, such as ER-specific tracers [12].

Prostate-specific membrane antigen (PSMA) radiopharmaceuticals have gained significant traction in the last decade [13]. These tracers target a seven-pass protein, which is usually overexpressed on the surface of prostate cancer cells. However, several studies have detected significant PSMA uptake in other cancer forms, such as hepatocellular carcinoma or thyroid neoplasms [14,15]. These studies have highlighted significant PSMA expression on the tumoral neovasculature, which could represent a target for diagnostic PET tracers and theranostic radiopharmaceuticals such as [^177^Lu]Lu-PSMA-617.

These concepts are particularly intriguing in the BC scenario, given the high death toll of this disease worldwide and the lack of therapeutic alternatives in the advanced/undifferentiated setting [16,17]. In this review, we aimed to gather the available evidence on the utility of PSMA-targeted PET/CT in determining the disease extent in patients encompassing different BC subtypes.

## 2. Materials and Methods

### 2.1. Protocol

This systematic review was performed according to a predefined protocol [18], and the “Preferred Reporting Items for a Systematic Review and Meta-Analysis” (PRISMA 2020 statement) was employed as a guide in its development [19]. The complete PRISMA checklist is in the Supplementary Material (Table S1). Pre-registering was not carried out. As an initial inquiry, our team posed the following question: can PSMA-targeted PET imaging be beneficial in managing breast malignancies? The literature review used the Population, Intervention, Comparator, Outcomes (PICO) framework. The study eligibility criteria were established as follows: patients confirmed to have BC (Population) who underwent PSMA-targeted PET with any PSMA radioligand (Intervention), either compared or not with standard imaging (Comparator). The outcomes investigated included the diagnostic performance of PSMA-guided PET imaging in BC, the reported uptake of PSMA radioligands in BC primitive and metastatic lesions, and the comparison between the currently employed instrumental investigation and this innovative diagnostic technique. The literature search, study selection, and quality assessment were conducted independently by two reviewers (A.R. and G.T.). An online consensus meeting resolved any discrepancies among the reviewers.

### 2.2. Literature Search Strategy, Information Sources, and Eligibility Criteria

After defining the review question, two authors (A.R. and G.T.) independently searched PubMed/MEDLINE, Embase, and Cochrane Library for studies on PSMA-targeting PET’s diagnostic accuracy in BC. The authors also searched clinicaltrials.gov for relevant trials. A search algorithm using “PSMA” and “breast” was applied. No language or publication-year limitations were imposed. The references of included studies were further examined for additional acceptable publications to refine the research. ClinicalTrials.gov was searched for ongoing trials. The literature search was last updated on 3 September 2024.

This systematic review included PSMA-targeted PET clinical trials for BC staging and restaging. Reviews, letters, comments, editorials, case reports or short case series (papers enrolling less than 5 cases), and original papers concerning other fields of interest (including pre-clinical investigations) were excluded from the systematic review (qualitative analysis). Study reports in languages other than English were excluded.

### 2.3. Study Selection Process and Data Collection

The titles and abstracts of the collected papers were reviewed separately by two authors (A.R. and G.T.) according to the specified requirements for inclusion or exclusion and the literature search strategy. They also provided reasons for their choices based on the bibliographic databases. The same authors (A.R. and G.T.) independently gathered all the relevant studies to ensure impartiality and extracted data from all the available sources, including full text, tables, figures, and Supplemental Material. Data from each study included in the systematic review were extracted in the following manner: general information provided in the study: authors, publication year, country, study design, and funding sources; patient characteristics: sample size, age, clinical setting, and other diagnostic imaging; index text characteristics: the type of PSMA radioligand used, the hybrid imaging protocol, patient preparation, radiopharmaceutical administered activity, uptake time between PSMA radioligand administration and image acquisition, and the protocol for image analysis. Additionally, when available, data on the diagnostic performance of PSMA-targeted PET in BC were analysed on a per-patient or per-lesion basis.

### 2.4. Quality Assessment (Risk of Bias Assessment)

The chosen method for evaluating the risk of bias in individual studies and its relevance to the review question was QUADAS-2, a tool designed for assessing quality in diagnostic test accuracy studies. Three reviewers, A.R., D.A. and G.T., conducted independent assessments of the quality of the studies included in the systematic review and meta-analysis. The analysis assessed four domains (patient selection, index test, reference standard, and flow and timing) to individuate potential bias and evaluated three fields (patient selection, index test, and reference standard) for applicability. A virtual consensus meeting resolved all reviewer disagreements regarding the quality assessment.

## 3. Results

### 3.1. Literature Search Results

**Figure 1:**
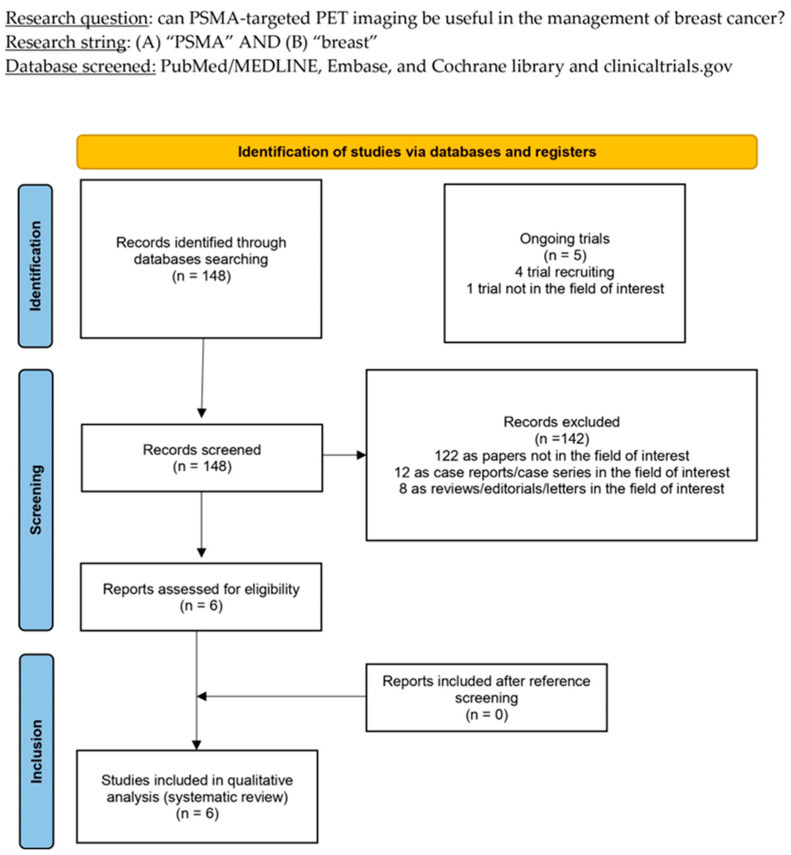
PRISMA flow-chart summarising study selection process. PRISMA: Preferred Reporting Items for Systematic Reviews and Meta-Analysis.

After conducting a comprehensive literature search, 148 records were found. Based on the details provided in the Section 2, a thorough evaluation was performed on all publications to determine their eligibility. Specific criteria were established to determine which documents would be included or excluded. As a result, 142 documents were deemed ineligible due to their irrelevance to the topic of interest or because they were either case reports or reviews. After careful evaluation, the six remaining studies were deemed appropriate for inclusion in the systematic review, specifically for qualitative synthesis [20,21,22,23,24,25]. Upon reviewing the references of the included articles, it was determined that no further studies met the criteria for inclusion in the systematic review. Figure 1 provides an overview of the selection process followed in the studies.

### 3.2. Study Characteristics

The six studies meeting the criteria for inclusion in the systematic review (qualitative analysis), which included 153 patients, are thoroughly analysed in Table 1, Table 2 and Table 3. The selected studies were published from 2017 to 2024. Four included papers accounted for a prospective design [20,22,23,25], while the remaining two studies retrospectively analysed their casuistries [21,24]. Every trial but one was single-centre [21,22,23,24,25]; moreover, only one of the included papers disclosed financing in its text [25]. General data concerning the included studies are reported in Table 1.

The number of patients ranged from 10 to 42, and their average age ranged from 45 to 61. The enrolled patients were only female in all the studies included in the systematic review. In all the articles included except for one, the index test was employed for staging or restaging BC patients; in the remaining paper, PSMA-targeted PET/CT was used for staging newly diagnosed BC patients. All studies included [^18^F]FDG PET as a Comparator [20,21,22,23,24,25]; however, two studies added conventional imaging (CT and bone scan) as a Comparator of the index test. Table 1 presents all patient characteristics.

All the studies except one administered [^68^Ga]Ga-PSMA-11 as a PSMA-targeting compound for PET imaging [20,21,22,23,24], whereas the remaining one used [^18^F]PSMA-1007 [25]. The patients received an activity ranging from 74 to 350 MBq [20,21,22,23,24]; in one study, an activity of 3 MBq/kg [25] was reported. The uptake time was about one hour for all studies [20,21,22,23,24,25]. A qualitative and semiquantitative evaluation of PET imaging was performed in all cases [20,21,22,23,24,25]. Only one study used the “prostate cancer molecular imaging standardised evaluation” (PROMISE) to evaluate the uptake areas qualitatively [24]. Most studies used SUVmax as a PET metric [21,22,23,24,25]; Sathekge et al. used SUVmean [20]. Table 2 synthesises the technical aspects concerning PSMA-targeted PET imaging execution in the included studies.

**Table 2:** Technical characteristics of the index test.

Authors [Ref.]	Tracer	Hybrid Imaging	Tomograph	Administered Activity	Uptake Time (min)	Image Analysis
Sathekge et al. [20]	[^68^Ga]Ga-PSMA-11	PET/CT	Not reported	Not reported	Not reported	Qualitative, Semiquantitative (SUV_mean_)
Medina-Ornelas et al. [21]	[^68^Ga]Ga-PSMA-11	PET/CT	Biograph mCT (Siemens)	Range: 88–240 MBq	59	Qualitative, Semiquantitative (SUV_max_)
Arslan et al. [22]	[^68^Ga]Ga-PSMA-11	PET/CT	mCT 20 ultraHD LSO (Siemens)	Range: 77–350 MBq	60	Qualitative, Semiquantitative (SUV_max_)
Mushtaq et al. [23]	[^68^Ga]Ga-PSMA-11	PET/CT	Discovery MV690 (GE)	Range: 107–958 MBq	60	Qualitative, Semiquantitative (SUV_max_)
Parghane et al. [24]	[^68^Ga]Ga-PSMA-11	PET/CT	GEMINI TF (Philips)	Range: 74–111 MBq	60	Qualitative (PROMISE V2), Semiquantitative (SUV_max_)
Andryszak et al. [25]	[^18^F]PSMA-1007	PET/CT	Discovery IT	3MBq/kg	60	Qualitative, Semiquantitative (SUV_max_, TBR)

Legend: CT: computed tomography; PSMA: prostate-specific membrane antigen; MBq: MegaBecquerel; PET: positron emission tomography; PROMISE V2: prostate cancer molecular imaging standardised evaluation version 2; SUV: standardised uptake value; TBR: target-to-background ratio.

### 3.3. Risk of Bias and Applicability

**Figure 2:**
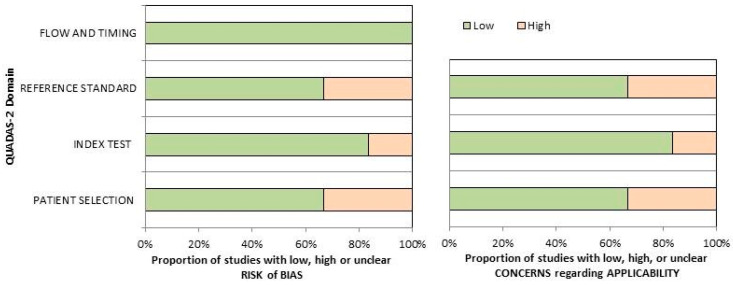
Quality assessment according to QUADAS-2 tool. The papers included in the systematic were classifie as high risk or low risk of bias or applicability concerns for distinct domains listed in the ordinate axis. The abscissa axis shows the percentage of studies.

Figure 2 presents the comprehensive evaluation of the risk of bias and issues regarding the applicability of the included publications as per QUADAS-2. The quality evaluation of the research reviewed indicated a moderate risk for biases in the area of “reference standard” and “patient selection”. The first can be attributed to the inhomogeneous Comparator selection for different patients in the same study; the latter can be attributed to the small sample size reported and the inclusion of patients from various clinical settings in individual trials.

### 3.4. Results of Individual Studies

The overall performance of PSMA-targeted PET/CT in detecting BC lesions was variable across the included papers, in per-patient- and per-lesion-based analyses in all the investigated clinical settings, without significant differences between the staging or restaging setting [20,21,22,23,24,25]. This finding reflects the high heterogeneity of accuracy values extracted from the different studies and in various sites of disease (primary, lymph nodes, distant metastases). Overall, the detection rate (DR) ranged between 66.6% and 99% in the primary lesions and between 43% and 81% for the secondary lesions. The metastatic tumour sites were the lung, bone, liver, brain, peritoneum, and adrenal glands [20,21,22,23,24,25]. Compared to standard imaging, PSMA-PET showed an inferior number of metastatic lesions (except for brain metastases), and could not affect patients’ management [20,21,22,23,24,25].

PSMA-targeting radiopharmaceuticals exhibited variable uptake across the primary tumour, local recurrence, and nodal and distant metastases, showing overall low values. However, it consistently measured higher than the background in most studies. It demonstrated a very high tumour-to-background ratio in brain metastases, typically not detectable with [^18^F]FDG PET/CT due to high brain metabolic activity [20,21,22,23,24,25].

Concerning BC subtypes, two studies observed higher uptake in less differentiated forms (TN and ER/PR^-^ HER2+ subtypes) [21,24]. On the other hand, one paper found no significant difference in progesterone receptor-positive and -negative lesions [20].

One study included an immunohistochemical analysis in biopsied primary or metastatic BC [22]. Twenty-nine tumour lesions exhibited Clau1 expression in 86% of the biopsied lesions, and 48% were PSMA-positive. No statistically significant link was revealed between the Ki-67 index and scores for PSMA, Clau 1, Clau 4, and Clau 7. Positive correlations were observed between the mean primary lesion uptake and the lesion’s axial diameter. Conversely, a negative connection was found between the mean primary lesion PSMA avidity and the Ki-67 index. However, there was no significant correlation between in vivo PSMA expression, as evaluated by PET imaging, and nuclear grade, claudin expression, and nodal and distant metastases.

Finally, one study qualitatively evaluated the PSMA-targeting radiopharmaceutical uptake of metastatic lesions to assess if they were suitable for PSMA-targeted radioligand therapy (RLT) using the “prostate cancer molecular imaging standardised evaluation” (PROMISE) as a benchmark to determine which patients could benefit from this treatment [24,26]. The authors observed a higher PSMA radioligand uptake (PROMISE score 2 or 3) in individuals with TN and ER/PR-HER2+ hormonal statuses on qualitative PSMA; moreover, they performed a region-based analysis of hormonal statuses and their association with PSMA expression for PRLT eligibility, observing a significant correlation in breast for triple-positive tumours and in axillary, chest wall, and skeleton regions for TN BCs. This study concluded that a modest number of BC patients were suitable for PSMA-targeted RLT and that PSMA-targeted PET/CT imaging could be beneficial for BC patients as a “theranostics selector” in a subset of BC patients, particularly those with TN and hormone receptor-negative/HER2+ hormonal statuses for PSMA-targeting RLT. Table 3 summarises the main results of the included studies.

**Table 3:** PET and diagnostic parameters of the included studies.

Authors [Ref.]	PET Metrics	Lesions Site	Diagnostic Performance		Outcome
			Per Patient	Per Lesion	
Sathekge et al. [20]	SUV_mean_ Primary: 2.45 Metastases: 6.8	Lymph node Lung Bone	N.A.	DR: 84%	In vivo PSMA assessment in BC shows valuable heterogeneity.
Medina-Ornelas et al. [21]	N.A.	Lymph node Soft tissues Bone	Primary DR: 99% Lymph nodes DR: 81% Metastases DR: 71% Sensitivity: 84% Specificity: 91.8%	N.A.	PSMA-PET showed high DRs in staging TN and HER2+ BC patients, suggesting that PSMA could be a suitable target for RLT in selected BC patients.
Arslan et al. [22]	SUV_max_ Primary: 6.6 Lymph nodes: 3.8 Metastases: 6.0	Lymph node Liver Lung Bone Brain	N.A.	DR Primary: 88.1%	PSMA-PET showed fewer lesions than FDG-PET. However, PSMA may be a potential RLT target in selected BC patients.
Mushtaq et al. [23]	SUV_max_ Primary: 2.3 Lymph nodes: 2.6 Metastases: 5.5	Lymph node Bone Peritoneum	DR Lymph nodes: 80%% DR Metastases: 43%%	DR Primary: 66.6%	There are no differences in DR between standard-of-care imaging and PSMA-PET.
Parghane et al. [24]	N.A.	Lymph node Liver Lung Bone	Sensitivity: 65.5% Specificity: 93.3%	N.A.	Based on qualitative uptake metrics, some BC patients would be eligible for RLT.
Andryszak et al. [25]	SUV_max_ Primary: 6.0 Lymph nodes: 4.3 Lung Metastases: 3.1	Lymph node Lung Liver Bone Brain Adrenal gland	N.A.	N.A.	PSMA-PET showed similar performance compared to FDG-PET. It could highlight brain lesions, which are invaluable with FDG-PET.

## 4. Discussion

In the present study, we tried synthesising the current status quo of the research on PSMA PET in BC. The findings offer two main messages: First, PSMA-targeted PET seems to have good diagnostic capabilities, particularly when applied to aggressive BC entities such as triple-negative or HER-positive forms. In lobular BC, the technique appears to be less sensitive; however, this issue has been reported by [^18^F]FDG PET/CT studies as well [12]; in this entity, molecular imaging of the oestrogen receptors appears to be the best PET technique. The second point is that even though the tracer uptake intensity is consistently higher than the background, it does not reach the level usually required to consider a theranostic approach in prostate cancer (i.e., equal or higher than the liver/spleen uptake). However, these findings alone do not rule out the possibility of a radionuclide treatment with PSMA-targeting radiopharmaceuticals in metastatic BC; other factors, such as the tracer kinetics, are bound to impact the delivered dose. Targeted trials should investigate this aspect.

The study confirms the relevance of the PSMA-guided approach in imaging and theranostics [27,28]; furthermore, it confirms that, with this receptor expressed on the tumour neovasculature, it will be apt to image many cancer forms other than prostate cancer, including clear cell renal carcinoma, thyroid cancer, and hepatocellular carcinoma [17,29,30]. Immunohistochemistry studies observed how PSMA expression could regulate tumour cell invasion and neoangiogenesis by transducing integrin signals in the endothelium [31]. Although the available evidence is still preliminary, and the studies screened employed different methods for estimating the diagnostic accuracy, it was comparable with the reference standard in most cases with hormone-receptor-negative/HER-2 positive and TN BC patients showing a higher number of lesions and greater PSMA expression (assessed both in vitro and in vivo) compared to hormone receptor-expressing malignancies [20,21,22,23,24,25]. The findings provided by the included studies confirm the great degree of heterogeneity that can be found across the different BC forms and even within a single subject bearing metastases in various organs. The hormone-receptor-negative/HER-2 positive and TN BC subtypes are the most aggressive forms, so, unsurprisingly, neo-endothelium shows higher PSMA expression in these subtypes than in less aggressive variants [24].

The therapeutic choices for TN BC patients are limited because of the lack of oestrogen, progesterone, and HER2 receptors on the tumour cell membrane. Several ongoing studies are investigating the possible influence of the TNBC microenvironment and identifying molecular subgroups that would benefit from successful immunotherapy [32]. In this setting, the assessment of potential molecular targets is a crucial step to allow the development of new therapeutic strategies to contain tumour growth and render metastatic TN BCs a chronic disease. In 2019, Morgenroth et al. demonstrated for the first time in vitro and in vivo the potential of targeting neovasculature-associated PSMA by radiolabelled PSMA-targeting radiopharmaceuticals as a potential therapeutic option in TN BC mice [33]. More recently, Heesch and colleagues performed an in vivo evaluation of [^177^Lu]Lu-PSMA-imaging I&T imaging and therapy in TN BC mice, comparing a single dose and a fractionated-dose approach, and observed that RLT could inhibit tumour growth and improve survival rates in the models acting as an anti-angiogenic radiotherapy [34]. Only one case reports the administration of PSMA-targeted RLT in a TN BC human patient [35]. According to the authors, the RLT was well tolerated by the patient without significant side effects; however, she showed severe progression four weeks after the second cycle, and no further administrations were applied. Based on the described background and the PSMA expression demonstrated by several studies, prospective multicentre studies are needed to assess if this therapy might become an affordable option for metastatic TN BC patients with progressive disease after standard-of-care treatments. However, the possibility of a real therapeutic effect of PSMA appears unlikely a priori, given the uptake mechanism. Recently, Fluorine-oestradiol ([^18^F]FES) PET/CT has been introduced as an efficacious imaging modality for patients with metastatic ER-expressing BC to evaluate the ER expression in loco-regional and distant metastases [36]. Although [^18^F]FES PET/CT has garnered considerable interest as a non-invasive method for predicting the efficacy of hormone-blocking treatments, its efficacy in diagnostic performance is comparable to [^18^F]FDG PET/CT, according to the available literature [36]. On the other hand, PSMA-targeted PET/CT showed more reliable results in patients with negative hormone receptor status; this statement let us postulate that PSMA-targeted PET/CT and [^18^F]FES might have a complementary role in BC patients. However, considering the absence of head-to-head studies comparing these novel tracers in BC patients, prospective hormone-status-based dual-tracer trials are needed to validate this hypothesis. A past systematic review examined the possible applications of PSMA-targeted PET/CT in BC patients [37]. However, it included case reports that have poor evidence quality and are influenced by publication bias. The main goal of our systematic review was to perform a current literature search per the PRISMA specifications and use a rigorous approach, employing appropriate criteria. Therefore, our literature review included the presentation and discussion of Supplementary Material.

The main limitation of this systematic review lies in the limited number of included studies and their patient selection criteria, which included the enrolment and analysis of patients with different BC subtypes. Moreover, our analysis revealed a notable variation among the gathered studies regarding the reference standard utilised to establish the clinical efficacy of the suggested diagnostic approach, which could be a potential source of bias.

## 5. Conclusions

Based on the evidence from the single studies and the absence of concrete modifications in BC patient management guided by PSMA-targeted PET/CT, this novel diagnostic examination cannot be suggested as an alternative to standard-of-care imaging. Nevertheless, PSMA-guided imaging might be a valuable instrument for selecting metastatic BC patients who could benefit from PSMA-targeting RLT; in this setting, more prospective trials are warranted to assess if this novel therapeutic strategy is suitable to enter clinical practice.

## Figures and Tables

**Table 1:** Patiants’ characteristics.

Authors [Ref.]	Year	Country	Study Design	Funding Sources	Sample Size	Mean/Median Age	Clinical Setting (No. Patients)	Histopathological BC Subtypes (No. Patients)	Receptor Status	Comparator
Sathekge et al. [20]	2017	South Africa/Belgium	P/M	N	19	Mean: 45	9 Staging 10 Restaging	13 Ductal 2 Lobular 1 Neuroendocrine 3 Unknown	6 PgR+ 7 PgR+ 6 PgR-unknown	CT, BS, [^18^F]FDG PET
Medina-Ornelas et al. [21]	2020	Mexico	R/S	N	21	Mean: 51	21 Staging	N.A.	4 LUM-A 4 LUM-B HER2+ 2 LUM-B HER2− 6 HER2 5 TN	[^18^F]FDG PET
Arslan et al. [22] *	2023	Turkey	P/S	N	42	Mean: 49.8	36 Staging 6 Restaging	N.A.	47 TN	[^18^F]FDG PET
Mushtaq et al. [23]	2024	U.S.A.	P/S	N	20	Mean: 55.5	7 Staging 13 Restaging	20 Lobular	16 ER+PgR+HER2- 2 ER+PgR+HER2+ 2 ER+PgR-HER2-	CT, BS, [^18^F]FDG PET
Parghane et al. [24]	2024	India	R/S	N	41	Median: 54	11 Staging 30 Restaging	N.A.	15 LUM-A 6 LUM-B HER2− 5 HER2 15 TN	[^18^F]FDG PET
Andryszak et al. [25]	2024	Poland	P/S	Y **	10	Median 62	1 Staging 9 Restaging	N.A.	10 TN	[^18^F]FDG PET

* Some patients had more than one breast lesion. ** Funded by the Ministry of Science and Higher Education of the Republic of Poland. Legend: P: prospective; R: retrospective; M: multicentre; S: single-centre; BS: bone scan; BC: breast cancer; CT: computed tomography; ER: oestrogen receptor; FDG: fluorodeoxyglucose; LUM-A: luminal A; LUM-B: luminal B; N.A.: not available; PET: positron emission tomography; PgR: progesterone receptor; TN: triple-negative.

## Supplementary Materials

The supporting Information can be downloaded at https://www.mdpi.com/article/10.3390/ijms252111413/s1. Reference [38] is cited in the Supplementary Material.
